# Supplementary material for: A Critical View of the Application of the APEX Software (Aqueous Photochemistry of Environmentally-Occurring Xenobiotics) to Predict Photoreaction Kinetics in Surface Freshwaters
Source: Molecules. 2019 Dec 18;25(1):9. doi: 10.3390/molecules25010009 (PMC7017383; doi:10.3390/molecules25010009)
Supplement: Supplementary file 1 [file molecules-25-00009-s001.zip › 3.2.4_gcc-4.4.0.zip!/3.2.4_gcc-4.4.0/3.2.4_gcc-4.4.0/doc/octave/html/interpreter/Manipulating-Classes.html]

Manipulating Classes - Untitled


Next: Indexing Objects,
Previous: Creating a Class,
Up: Object Oriented Programming


---

### 33.2 Manipulating Classes

There are a number of basic classes methods that can be defined to allow
the contents of the classes to be queried and set. The most basic of
these is the `display` method. The `display` method is used
by Octave when displaying a class on the screen, due to an expression
that is not terminated with a semicolon. If this method is not defined,
then Octave will printed nothing when displaying the contents of a class.

— Function File: **display** (a)  
> Display the contents of an object. If a is an object of the
> class "myclass", then `display` is called in a case like
>
> ```
>           myclass (...)
> ```
>
> where Octave is required to display the contents of a variable of the
> type "myclass".
>
>
>
> **See also:** class, subsref, subsasgn.

An example of a display method for the polynomial class might be

```
```
     function display (p)
       a = p.poly;
       first = true;
       fprintf("%s =", inputname(1));
       for i = 1 : length (a);
         if (a(i) != 0)
           if (first)
             first = false;
           elseif (a(i) > 0)
             fprintf (" +");
           endif
           if (a(i) < 0)
             fprintf (" -");
           endif
           if (i == 1)
             fprintf (" %g", abs (a(i)));
           elseif (abs(a(i)) != 1)
             fprintf (" %g *", abs (a(i)));
           endif
           if (i > 1)
             fprintf (" X");
           endif
           if (i > 2)
             fprintf (" ^ %d", i - 1);
           endif
         endif
       endfor
       if (first)
         fprintf(" 0");
       endif
       fprintf("\n");
     endfunction
```
```

Note that in the display method, it makes sense to start the method
with the line `fprintf("%s =", inputname(1))` to be consistent
with the rest of Octave and print the variable name to be displayed
when displaying the class.

To be consistent with the Octave graphic handle classes, a class
should also define the `get` and `set` methods. The
`get` method should accept one or two arguments, and given one
argument of the appropriate class it should return a structure with
all of the properties of the class. For example

```
```
     function s = get (p, f)
       if (nargin == 1)
         s.poly = p.poly;
       elseif (nargin == 2)
         if (ischar (f))
           switch (f)
             case "poly"
               s = p.poly;
             otherwise
               error ("get: invalid property %s", f);
           endswitch
         else
           error ("get: expecting the property to be a string");
         endif
       else
         print_usage ();
       endif
     endfunction
```
```

Similarly, the `set` method should taken as its first argument an
object to modify, and then take property/value pairs to be modified.

```
```
     function s = set (p, varargin)
       s = p;
       if (length (varargin) < 2 || rem (length (varargin), 2) != 0)
         error ("set: expecting property/value pairs");
       endif
       while (length (varargin) > 1)
         prop = varargin{1};
         val = varargin{2};
         varargin(1:2) = [];
         if (ischar (prop) && strcmp (prop, "poly"))
           if (isvector (val) && isreal (val))
             s.poly = val(:).';
           else
             error ("set: expecting the value to be a real vector");
           endif
         else
           error ("set: invalid property of polynomial class");
         endif
       endwhile
     endfunction
```
```

Note that as Octave does not implement pass by reference, than the
modified object is the return value of the `set` method and it
must be called like

```
     p = set (p, "a", [1, 0, 0, 0, 1]);
```

Also the `set` method makes use of the `subsasgn` method of
the class, and this method must be defined. The `subsasgn` method
is discussed in the next section.

Finally, user classes can be considered as a special type of a
structure, and so they can be saved to a file in the same manner as a
structure. For example

```
     p = polynomial ([1, 0, 1]);
     save userclass.mat p
     clear p
     load userclass.mat
```

All of the file formats supported by `save` and `load` are
supported. In certain circumstances, a user class might either contain
a field that it makes no sense to save or a field that needs to be
initialized before it is saved. This can be done with the
`saveobj` method of the class

— Function File: b = **saveobj** (a)  
> Method of a class to manipulate an object prior to saving it to a file.
> The function `saveobj` is called when the object a is saved
> using the `save` function. An example of the use of `saveobj`
> might be to remove fields of the object that don't make sense to be saved
> or it might be used to ensure that certain fields of the object are
> initialized before the object is saved. For example
>
> ```
>           function b = saveobj (a)
>             b = a;
>             if (isempty (b.field))
>                b.field = initfield(b);
>             endif
>           endfunction
> ```
>
>
>
>
> **See also:** loadobj, class.

`saveobj` is called just prior to saving the class to a
file. Likely, the `loadobj` method is called just after a class
is loaded from a file, and can be used to ensure that any removed
fields are reinserted into the user object.

— Function File: b = **loadobj** (a)  
> Method of a class to manipulate an object after loading it from a file.
> The function `loadobj` is called when the object a is loaded
> using the `load` function. An example of the use of `saveobj`
> might be to add fields to an object that don't make sense to be saved.
> For example
>
> ```
>           function b = loadobj (a)
>             b = a;
>             b.addmissingfield = addfield (b);
>           endfunction
> ```
>
>
>
>
> **See also:** saveobj, class.
